# Less invasive treatment for broad ligament hernia: A case report

**DOI:** 10.1016/j.ijscr.2020.07.012

**Published:** 2020-07-15

**Authors:** Tetsuo Sugishita, Shunsuke Kato, Aoi Ishikawa, Hidenori Takahashi, Yasuyuki Kawachi

**Affiliations:** Department of Surgery, Musashino Red Cross Hospital, 1-26-1, Kyounanchou, Musashino-city, Tokyo, Japan

**Keywords:** Uterus, Bowel, Obstruction, Laparoscopic surgery

## Abstract

**Introduction:**

Among diseases that cause small bowel obstruction, internal hernia may result in severe outcomes. Therefore, emergency laparotomy has been often performed, but it may be invasive. We experienced a case of a broad ligament hernia of the uterus, which is relatively rare among the cases of internal hernia that were treated by less invasive laparoscopic surgery.

**Case presentation:**

A 71-year-old woman came to our hospital because of abdominal pain and vomiting. Abdominal computed tomography (CT) revealed a broad ligament hernia of the uterus without intestinal ischemia. Intestinal decompression was done by inserting an ileus tube (® Long Intestinal Tube, CLINY), followed by elective laparoscopic surgery.

**Discussion:**

If intestinal ischemia can be ruled out, a less invasive laparoscopic surgery may be performed after intestinal decompression. Further, by detecting the cause of internal hernia, more safe and smooth surgery can be performed. These findings highlighted the importance of accurate CT diagnosis.

**Conclusion:**

In order to perform laparoscopic surgery for internal hernia, evaluation of the presence or absence of intestinal ischemia and detection of the cause of obstruction by preoperative abdominal CT are important.

## Highlights

• We experienced a case of a broad ligament hernia of the uterus, which is relatively rare among the cases of internal hernia that were treated by less invasive laparoscopic surgery.
• If intestinal ischemia can be ruled out, a less invasive laparoscopic surgery may be performed after intestinal decompression.
• Making a diagnosis of broad ligament hernia of the uterus by CT can lead to easier and safer laparoscopic surgery and shorter operation time.

## 1 Introduction

Small bowel obstruction is one of the most common diseases to treat for surgeons. In particular, small bowel obstruction secondary to internal hernia may cause intestinal ischemia and result in severe outcomes [1]. Emergency laparotomy is often performed for internal hernia, but operating on a patient before evaluating risks may be invasive. Therefore, it is important to assess the necessity for emergency surgery. If intestinal ischemia is ruled out, less invasive and safe surgery may be performed after correcting the dehydration and evaluating patient risks. We encountered a case of a broad ligament hernia of the uterus, which is relatively rare among the cases of internal hernia and can be treated less invasively by elective laparoscopic surgery after intestinal decompression. This surgery was conducted at a general hospital, and this work has been reported in line with the SCARE criteria [2].

## 2 Presentation of case

A 71-year-old woman came to our hospital because of persistent abdominal pain and vomiting for two days. The patient had a normal pregnancy history and has undergone right ovarian cyst surgery.

**Fig. 1:**
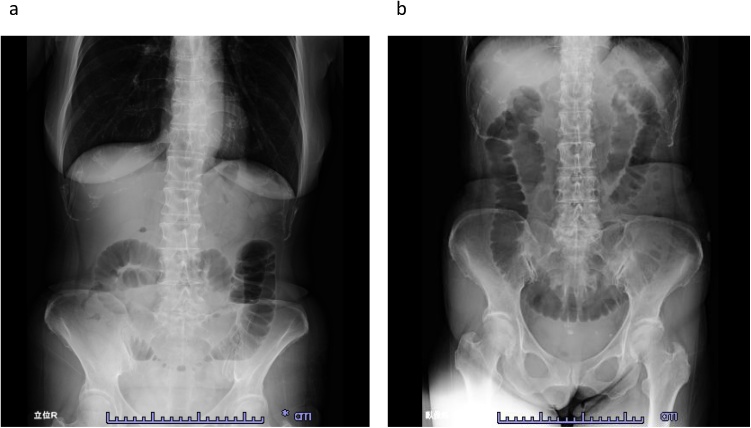
X-ray of the abdomen. a: Upright position. b: Seated position.

**Fig. 2:**
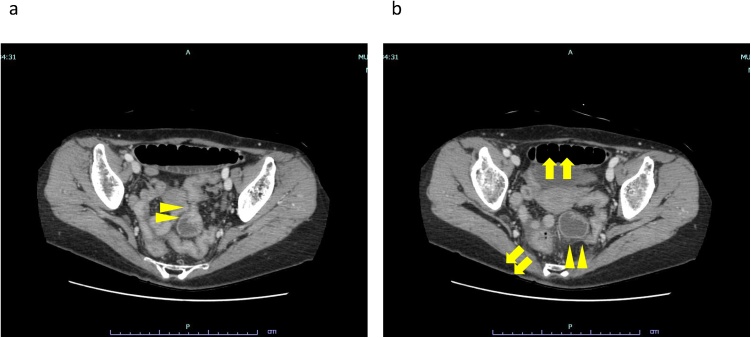
abdominal CT. a: A closed loop with the broad ligament of the uterus as the herniaorifice. b: A small bowel dilation in the pelvis (arrowheads). The uterus was slightly shifted ventrally and the recto-sigmoid shifted dorso-laterally (arrows).

Physical examination revealed bloating without tenderness and no fever. The white blood cell count was 17,400/μL, C-reactive protein was 0.6 mg/dL, and blood urea nitrogen was 39.7 mg/dL. X-ray of the abdomen showed small bowel dilation (Fig. 1). Abdominal computed tomography (CT) showed dilated small bowels in the pelvis and formation of a closed loop by invagination into the broad ligament of the uterus, which was the hernia orifice. The uterus was slightly shifted ventrally, and the rectosigmoid colon shifted dorsolaterally. No evidence of intestinal ischemia was observed (Fig. 2). Based on these findings, the patient was diagnosed as small bowel obstruction secondary to broad ligament hernia of the uterus and was admitted to the hospital immediately after ileus tube (® Long Intestinal Tube, CLINY) insertion. After intestinal decompression by intermittent continuous suction, laparoscopic surgery was performed six days after admission.

**Fig. 3:**
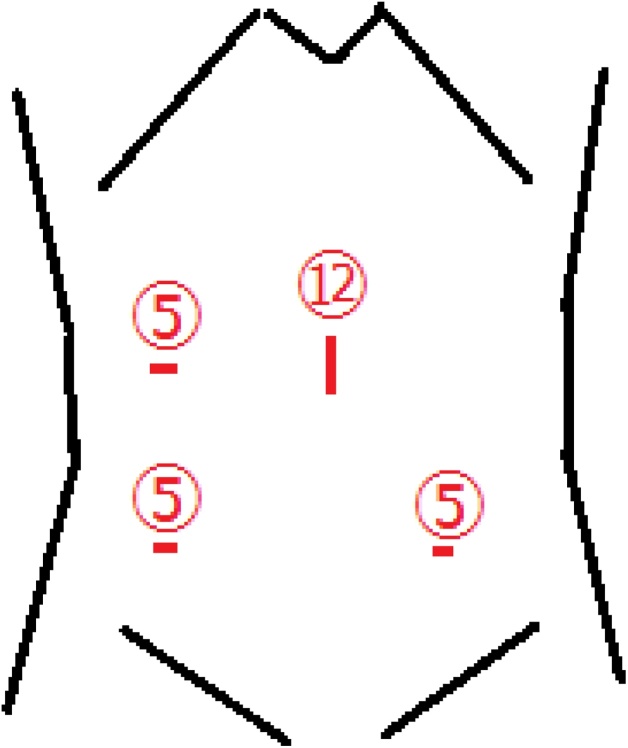
Position of the ports.

**Fig. 4:**
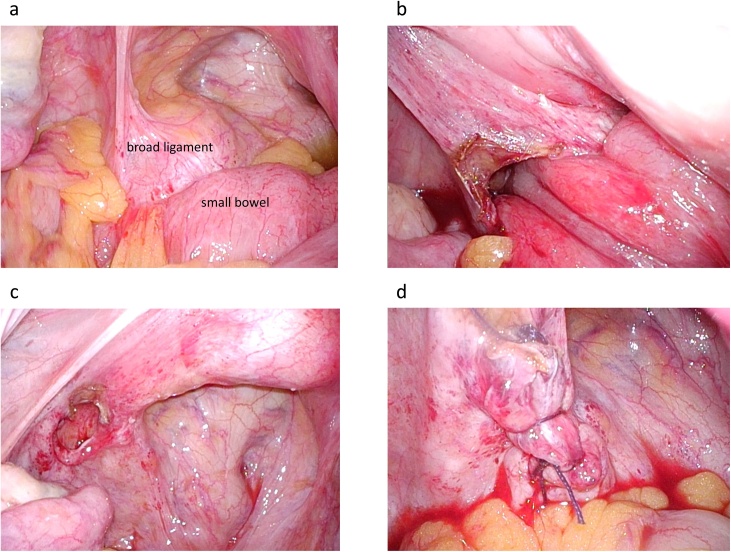
Pictures on surgery. a: The invaginating small bowel was observed in the left broad ligament of the uterus. b: Using an ultrasonic coagulation device, an incision was made on the broad ligament of the uterus to dilate the hernia orifice. c: The invaginating small intestine was released by traction. d: The hernia orifice was closed with a continuous suture using absorbent thread.

The surgery was performed by a resident of surgeon, board-certified general surgeon and board-certified laparoscopic surgeon. The surgery was done using four ports, including the navel, upper right abdomen, lower right abdomen, and lower left abdomen (Fig. 3). Because of sufficient decompression of the small bowel, there was no trouble inserting the surgical port and securing the operative field. After removing the sigmoid colon and the small bowel in the pelvis by pushing cephaladly, the invaginating small bowel was observed in the left broad ligament of the uterus. Using an ultrasonic coagulation device, an incision was made on the broad ligament of the uterus to dilate the hernia orifice. After releasing the invaginating small bowel by traction, the absence of ischemia or perforation of the invaginating small bowel was confirmed. The hernia orifice was closed with a continuous suture using absorbent thread (Fig. 4). The operation time was 80 min, and blood loss was 10 mL.

On postoperative day 4, defecation was observed and X-ray of the abdomen showed improvement of the small bowel dilation. There was no trouble after initiation of diet, and the patient was discharged on postoperative day 7. There is no recurrence for 2 years.

## 3 Discussion

Broad ligament hernia of the uterus is a state in which an abdominal organ invaginates into the broad ligament of the uterus. It is classified as a pouch type if it penetrates only the anterior lobe of the broad ligament of the uterus or as a fenestra type if penetrates the entire broad ligament of the uterus [3]. The typical patient is a middle-aged multiparous woman with no history of abdominal surgery [4]. Regardless of type, the first choice of treatment is surgery by either closing the defect with suture or surgical clips or by opening the defect [5].

Based on our experience on this patient who was treated less invasively, we would like to put emphasis on two key points. First, when the abdominal CT shows no intestinal ischemia and the pain is controlled, laparoscopic surgery may be possible after intestinal decompression. Second, if the diagnosis of broad ligament hernia of the uterus can be confirmed by abdominal CT, laparoscopic surgery may become a lot easier and safer, leading to shorter operation time.

Evaluation of the presence or absence of intestinal ischemia is important when deciding the treatment for internal hernias, including broad ligament hernia of the uterus. If the treatment of intestinal ischemia is delayed, the mortality rate may reach >50%, and prompt judgment is required [1]. However, the absence of evidence of intestinal ischemia may enable the safe performance of laparoscopic surgery, which is less invasive, by inserting an ileus tube and decompressing the intestine. Difficulty in securing a working space because of intestinal dilatation and visceral fat excess was reported to cause conversion of laparoscopic surgery to laparotomy in 22%–49% of cases of small bowel obstruction [6]. For the past 10 years in Japan, 2 of 39 cases of laparoscopic surgery for broad ligament hernia of the uterus was converted to laparotomy because of inadequate working space or difficulty of traction of the invaginating intestine. In those two cases, decompressing the intestine with as ileus tube was not preformed. In this present case, preoperative intestinal decompression contributed to safe and complete laparoscopic surgery.

Moreover, avoiding emergency surgery has more benefits, in addition to enabling laparoscopic surgery. Many patients with small bowel obstruction are dehydrated and are in unstable conditions. While decompressing the intestine, stabilizing the patient by hydration and evaluation of cardiac and pulmonary problems that could affect general anesthesia are possible.

Internal hernia was reported to account for 0.6%–5.8% of the cases of small bowel obstruction [7]. Furthermore, broad ligament hernia of the uterus accounts for 1.6%–5.0% of internal hernia cases and is relatively highly difficult to diagnose, because of its low frequency [8]. The following signs on CT are the characteristics of broad ligament hernia of the uterus: ① a cluster of dilated small bowel loops with air-fluid levels in the pelvic cavity and ② small bowel loops compressing the rectosigmoid colon dorsolaterally and the uterus ventrally [6]. In this case, it was possible to determine the cause of internal hernia based on the presence of these findings, thereby, leading to the performance of a safe and less invasive surgery. When treating patients with internal hernia, it would be helpful to keep these characteristics in mind.

The main causes of internal hernia are paraduodenal hernia (53%), pericecal hernia (13%), foramen of Winslow hernia (8%), and transmesenteric and transmesocolic hernia (6%) [9], all of which have been reported to be possibly treated by laparoscopic surgery [[10], [11], [12], [13]]. If a working space can be secured by decompressing the intestine, reduced port surgery may be selected, leading to a less invasive surgery. However, we should not hesitate to perform an emergency surgery if the abdominal symptoms and general condition get worse during intestinal decompression. Careful examination of the patient’s condition is important to determine whether a less invasive surgery can be selected.

## 4 Conclusion

For small bowel obstruction secondary to internal hernia, contrast-enhanced abdominal CT may be helpful to accurately evaluate the need for emergency surgery. If there is no need for emergency surgery, ileus tube decompression can be performed to achieve a less invasive surgery. Moreover, making a diagnosis of broad ligament hernia of the uterus by CT can lead to easier and safer laparoscopic surgery and shorter operation time.

## Declaration of Competing Interest

None.

## Sources of funding

The authors would like to thank Enago (www.enago.jp) for the English language review.

## Ethical approval

Ethical board approval is not necessary for case reports in our institution.

## Consent

Written informed consent was obtained from the patient for publication of this case report and accompanying images. A copy of the written consent is available for review by the Editor-in-Chief of this journal on request.

## Author contribution

Tetsuo Sugishita: data collection, writing the paper.

Shunsuke Kato: performed the surgery, review and editing.

Aoi Ishikawa: performed the surgery.

Hidenori Takahashi: performed the surgery.

Yasuyuki Kawachi: the supervisor.

## Registration of research studies

None.

## Guarantor

Tetsuo Sugshita.

## Provenance and peer review

Not commissioned, externally peer-reviewed.
